# CSRP2 suppresses colorectal cancer progression *via* p130Cas/Rac1 axis-meditated ERK, PAK, and HIPPO signaling pathways

**DOI:** 10.7150/thno.45674

**Published:** 2020-09-02

**Authors:** Lixia Chen, Xiaoli Long, Shiyu Duan, Xunhua Liu, Jianxiong Chen, Jiawen Lan, Xuming Liu, Wenqing Huang, Jian Geng, Jun Zhou

**Affiliations:** 1 Department of Pathology, Nanfang Hospital, Southern Medical University, Guangzhou 510515, China.; 2 Department of Pathology, School of Basic Medical Sciences, Southern Medical University, Guangzhou 510515, China.

**Keywords:** Colorectal cancer, CSRP2, Hippo, ERK, PAK

## Abstract

Metastasis is a major cause of death in patients with colorectal cancer (CRC). Cysteine-rich protein 2 (CSRP2) has been recently implicated in the progression and metastasis of a variety of cancers. However, the biological functions and underlying mechanisms of CSRP2 in the regulation of CRC progression are largely unknown.

**Methods:** Immunohistochemistry, quantitative real-time polymerase chain reaction (qPCR) and Western blotting (WB) were used to detect the expression of CSRP2 in CRC tissues and paracancerous tissues. CSRP2 function in CRC was determined by a series of functional tests *in vivo* and *in vitro*. WB and immunofluorescence were used to determine the relation between CSRP2 and epithelial-mesenchymal transition (EMT). Co-immunoprecipitation and scanning electron microscopy were used to study the molecular mechanism of CSRP2 in CRC.

**Results:** The CSRP2 expression level in CRC tissues was lower than in adjacent normal tissues and indicated poor prognosis in CRC patients. Functionally, CSRP2 could suppress the proliferation, migration, and invasion of CRC cells *in vitro* and inhibit CRC tumorigenesis and metastasis *in vivo*. Mechanistic investigations revealed a physical interaction between CSRP2 and p130Cas. CSRP2 could inhibit the activation of Rac1 by preventing the phosphorylation of p130Cas, thus activating the Hippo signaling pathway, and simultaneously inhibiting the ERK and PAK/LIMK/cortactin signaling pathways, thereby inhibiting the EMT and metastasis of CRC. Rescue experiments showed that blocking the p130Cas and Rac1 activation could inhibit EMT induced by CSRP2 silencing.

**Conclusion:** Our results suggest that the CSRP2/p130Cas/Rac1 axis can inhibit CRC aggressiveness and metastasis through the Hippo, ERK, and PAK signaling pathways. Therefore, CSRP2 may be a potential therapeutic target for CRC.

## Introduction

Colorectal cancer (CRC) is one of the most common malignant tumors worldwide and the fourth leading cause of cancer-related deaths. Metastasis and recurrence are the leading causes of death in patients with CRC 1. Although many altered pathways and abnormal genes have been identified, the precise molecular mechanisms underlying the tumorigenesis and metastasis of CRC are not entirely clear 1, 2. Therefore, there is an urgent need to explore the underlying molecular mechanisms in CRC recurrence and metastasis.

Cysteine-rich protein 2 (CSRP2) is a member of the LIM-only family of cysteine-rich proteins and contains two LIM domains 3. Members of this family include CSRP1, CSRP2, and CSRP3. These proteins have the LIM zinc finger domain, which is generally considered the entity of protein-protein interaction and plays a key role in a variety of biological processes, such as signal transduction, cytoskeleton formation, cell proliferation, and differentiation 4. The three CSRPs (CSRP1-3) are preferentially expressed in muscle cells and localized in the nucleus and cytoplasm. In the nucleus, CRPs can interact with transcription factors, such as MyoD, SRF, and GATA family proteins, to facilitate smooth (CRP1 and CRP2) or striated muscle (CRP3) differentiation 5. In the cytoplasm, they decorate filamentous actin structures and participate in cytoskeletal remodeling 6. However, the exact function of these proteins remains unclear.

CSRP2 is widely expressed in various tissues 7. Previous studies have shown that CSRP2 contributes to the assembly and/or maintenance of the actin backbone. It is involved in actin cytoskeleton rearrangement, and a lack of CSRP2 can promote the migration of vascular smooth muscle cells 8. CSRP2 has been studied in several tumors, including breast 9, gastric 10, hepatocellular cancers 11 and lymphoblastic leukemia 6 and is involved in the proliferation, migration and invasion of tumor cells. However, the CSRP2 role is controversial in different tumors, and its precise cellular function needs to be further elucidated. The expression of CSRP2 in CRC and its role in the growth and progression of CRC is unknown.

In this study, we found that the expression of CSRP2 was lower in CRC and correlated with poor prognosis. Further investigations revealed that CSRP2 could interact with p130Cas to suppress the activation of Rac1 and then inhibit EMT and metastasis of CRC *via* activating Hippo and inhibiting ERK and PAK-LIMK-cortactin signaling pathways. For the first time, our findings provide a deeper understanding of the functions and mechanisms of CSRP2 in CRC progression and may contribute to the development of new therapeutic targets for CRC.

## Materials and Methods

### Cell culture

FHC (CRL-1831) and 7 human CRC cell lines, including HCT116, SW620, HCT15, SW480, RKO, SW1116, and LoVo, came from the Cell Bank of Chinese Academy of Sciences (Shanghai). All cells were identified by (Str) map of short tandem repeats and passaged less than 6 months after resuscitation. All CRC cell lines were cultured in RPMI 1640 medium (Gibco, Gaithersburg, MD, USA) supplemented with 10% fetal bovine serum (Hyclone, Logan, USA) and 100 μm U/mL penicillin/streptomycin (Gibco) in a 37 °C humidified chamber containing 5% CO_2_.

### Tissue preparation

20 pairs of fresh CRC tissues and paired normal colorectal tissues from patients with primary CRC in Nanfang Hospital were analyzed by WB and qPCR. (Guangzhou, China). Tissue paraffin specimens of 215 patients with primary CRC were collected in Nanfang Hospital from 2000 to 2005. None of these patients received any chemotherapy or radiotherapy before the operation. The classification system (PTNM) determines the stage of the disease according to the size of the tumor, lymph node metastasis, and distant metastasis 12.

### Immunohistochemistry

The immunohistochemical (IHC) method was used to detect the expression of CSRP2 protein in 215 pairs of paraffin-embedded CRC tissues and matched adjacent normal colorectal tissues. The slices were heated, dewaxed, rehydrated, and put into sodium citrate buffer (pH buffer = 6.0) for antigen repair. The slide was then soaked in 3% hydrogen peroxide to inhibit endogenous peroxidase activity and sealed with sheep10% FBS/PBS. After rinsing three times, the slices were incubated overnight with the first antibody (rabbit anti-CSRP2, 1:500 dilution, # HPA045617; Sigma) at 4 °C. Slices were washed three times using PBS and then treated with a second antibody (anti-rabbit IgG 1:2000 diluted, # 7074; Cell Signal Technology, Danvers, MA, USA) for 40 min at 37 °C. After being stained with 3BI-3-diaminobenzidine (DAB), it was stained with hematoxylin, dehydrated, sealed, and observed.

IHC scores based on staining intensity and percentage of positive tumor cells were performed by two independent pathologists who did not know the clinical data. The staining intensity scores were 0 (negative), 1 (weak), 2 (medium) and 3 (strong). According to the percentage of the positive staining area of the specimen in the entire tumor area or the entire section, the staining degree was scored as 0 (0%), 1 (1-25%), 2 (26-50%), 3 (51-75%), 4 (76-100%). The sum of the strength and range scores is used as the final score of the CSRP2 (0-12). The final staining score of more than 7 was regarded as high expression and the score of less than 7 was regarded as the low expression of CSRP2.

### RNA extraction and qPCR

Trizol reagent (Invitrogen, Carlsbad, CA) was used to extract the total RNA of cells and fresh tissues. PrimeScript RT kit (Promega, Madison, WI, USA) was used to synthesize cDNA. qPCR was performed in ABI7500 Real-time PCR system (Applied Biosystems, Foster City, USA) using SYBR PreMix ex Taq benchmark (Takala, Dalian, China) . The expression of related genes was measured by the comparative 2-ΔΔCT method. The sequence of primers used to amplify CSRP2 is 5'- TCACGATGAAGAGATCTACTGC -3' (forward) and 5(f AGTGTTTGGATTTGTTGTAGGC-3' (reverse). GAPDH was used as an endogenous control.

### Western blotting analysis

Protein from cell or tissue lysates was separated by SDS-polyacrylamide gel electrophoresis (PAGE) and electro-transferred onto a polyvinylidene difluoride (PVDF) membrane (Pall Corp, Port Washington, NY). Then the membranes were blocked with 5% skimmed milk and incubated using primary antibodies anti-CSRP2 (1:800 dilution, Sigma), anti-GAPDH, anti-p130Cas, anti-p-p130Cas (Tyr410), anti-PAK, anti-p-PAK, anti-LIMK, anti-p-LIMK, anti-cortactin, anti-p-cortactin, anti-E-cadherin, anti-N-cadherin, anti-vimentin, anti-β-catenin (Cell Signaling Technology, Beverly, MA), anti-LATS1, anti-Yap (Proteintech), anti-ERK1/2, anti-p-ERK1/2 (Thr202/Tyr204), anti-p-Yap (Ser127) (Absci), anti-p-LATS1 (Thr1079/1041) (Bioss) at 4 °C. Then the membranes were incubated with appropriate secondary antibodies (anti-rabbit IgG, 1:3000 dilution, #7074; cell signaling). Enhanced chemiluminescence (Pierce, Rockford, 1L, USA) was used to detect the signal.

### Lentiviral and plasmid transduction and transfection

The lentiviral vector LV-CSRP2 (7 × 10^8^ TU/mL) (Gen Chem, Shanghai, China) containing puromycin resistance gene, luciferase gene, and CSRP2 overexpression gene was transfected into human adenocarcinoma cell lines HCT116 and RKO. At the same time, the blank vector (CON285; 5 × 10^8^ TU/mL) containing puromycin resistance gene and luciferase gene was used to transfect HCT116 and RKO as negative control (NC). Besides, lentiviral vector LV-CSRP2-RNAi (4 × 10^8^ TU/mL) (Gen Chem, Shanghai, China) containing puromycin resistance gene, luciferase gene, and CSRP2 silencing gene was transfected into human adenocarcinoma cell lines SW480 and HCT15. And the blank vector (CON206; 8 × 10^8^ TU/mL), containing puromycin resistance gene and luciferase gene, was used to transfect SW480 and HCT15 cells as negative control (NC). p130Cas-WT is a wild-type p130Cas plasmid constructed by inserting BCAR1 into vector PCDNA3.1 (You ming, Guangzhou, China). p130Cas-F15 is a human p130Cas mutant lentivirus (pSLenti-CMVBCAR1 (MUTs) -PGK-Puro-WPRE) by mutating all 15 tyrosines within the common motif YXXP in the P130Cas substrate domain (SD) to phenylalanine(Heyuan, Shanghai, China).POLO Deliver 3000 transfection reagents were purchased from Shanghai Remos Biotechnology Co., Ltd. Then, transduced cells were selected for 14 days with 0.6 mg/mL puromycin. Protein and mRNA of transfected cells were extracted for qPCR and WB analyses.

### Cell proliferation assay

1 × 10^3^ cells were seeded on 96-well plates and cultured for 24 h. 2-(2-Methoxy-4-nitrophenyl)-3-(4-nitrophenyl)-5-(2,4-disulfothenyl)-2H-tetrazolium salt (CCK-8, Dojindo, Rockville, USA) solution was added to each well and incubated for 2 h, then the absorbance of each well was measured at 450 nm with a Microplate Autoreader (Bio-Rad, Hercules, CA, USA). The experiment was repeated 3 times.

### Colony formation assay

The cells were inoculated on a 6-well plate (200 cells per well) and cultured for 2 weeks. Cells were fixed 30 minutes with 4% paraformaldehyde and dye with 1% Giemsa for 15 minutes. Count the number of colonies with cells more than 50. Three independent experiments were performed.

### Cell wound healing assay

1.2 × 10^6^ cells were inoculated on a 6-well tissue culture plate and incubated for 24 hours. Scratched wounds were made with 10 μl pipettes, and then the plate was washed for 3 times and cultured with serum-free RPMI 1640. The wound closure was observed at 0 h and 72 h, respectively. Images were taken to evaluate the level of cell migration. The ability of cell migration was quantified by measuring the distance between the advancing edges of cells in three randomly selected microscopic fields (× horizon 200) at each time point.

### Transwell invasion assay

The upper chamber of the Transwell chamber was pre-coated with Matrigel, and 2 × 10^5^ cells suspended in serum-free media were placed in the upper compartment of 8-μm-pore Transwells (BD Biosciences, San José, CA, USA) and 10% FBS was used as a chemo-attractant filled in the lower compartment. The cells were cultured at 37 °C for 2 days. The cells successfully passing through the 8-μm-hole were stained with 0.5% crystal violet for 15 minutes, and 5 visual fields (× fields 200) were randomly selected under the microscope and the cells were counted.

### Immunofluorescent (IF) assay

The cells were fixed with 4% paraformaldehyde for 15 minutes, washed three times, and then thoroughly penetrated with 0.5% Triton X Triton 100 for 15 minutes, then washed three times with PBS and sealed with 10% fetal bovine serum for 1 hour, then the first antibody was incubated overnight at 4 °C, and then incubated with green goat anti-rabbit antibody and red goat anti-rabbit antibody. After re-staining with 4-amino-6-diamino-2-phenylindole (DAPI, Sigma), the images were captured using an Olympus FV1000 confocal laser scanning microscope (Olympus USA, NY, USA) and Karl Zeiss inverted laser confocal microscope (LSM 880 with Airyscan, Germany). Statistical analysis results by using Image J software. The pseudopodia were observed by living staining with phalloidin (Cytoskeleton, PHDH1) staining and DAPI (nuclear staining) staining after the cells were fixed and permeated.

### Co-immunoprecipitation (co-IP) assay

A total of 1 × 10^6^ HCT116/CSRP2 cells (cells with overexpression of CSRP2 lentiviral) were inoculated in 10 cm plates. After 48 hours of culture, the cells were washed with cold PBS. The cold RIPA buffer is then added to the dish and the cells are scraped off with a pre-cooled scraper. Transfer the suspension to a new EP tube, oscillate for 15 min, and then centrifuge at 4 °C 1400 g for 15 min. Agarose beads were added to the protein (100 μL protein an agarose/1 mL protein). After shaking for 10 minutes at 4 °C, the sample was centrifuged at 1400 μg at 4 °C for 15 min, to remove protein G beads. The anti-CSRP2 (or anti-p130Cas) antibody was then rotated and incubated overnight at a temperature of 4 °C, and protein A was added the next day to capture the antigen-antibody complex. The complex was rotated and incubated overnight at 4 °C. After washing with washing buffer, the samples were centrifuged at 4 °C 1400 g for 2 min and repeated 6 times. The sample was boiled and denatured for 5 minutes and then washed with SDS-PAGE loading buffer. Finally, the samples were separated by SDS-PAGE and analyzed by WB.

### Pulldown assay for RhoA, Cdc42 and Rac1 activity

The active Rac1/Cdc42/RhoA (GTP binding form) was isolated according to the scheme provided by cytoskeletons “RhoA/Rac1/Cdc42 Activation Assay Combo Biochem Kit” (stock number: BK034, BK035, BK036). For the Rac1 activation inhibitor NSC23766 treatment, the cells were incubated in the medium containing 50uM NSC23766 for 24 hours. Cdc42, Rac1, and RhoA antibodies were used to detect the total amount and activity of total Cdc42, Rac1 and RhoA in the kit.

### Scanning electron microscopy (SEM)

Cover slides were added to the 24-well plate in advance to make the cells grow on the cover slides. When the fusion degree reaches about 50%, stop the culture of the sample, wash it with low temperature PBS, add 2.5% glutaraldehyde and fix it at room temperature for 1 hour. Then place it at 4 °C for 3 hours or overnight (pay attention to prevent drying). Then, it was dehydrated in increasing concentrations of acetone, and critical-point dried, fixed to stubs with colloidal silver, sputtered with gold using a MED 010 coater and examined under an S-3000 N scanning electron microscope (HITACHI Company, Japan) 13.

### Tumourigenesis in nude mice

BALB/C-nu/nu nude mice (3-4 weeks old) were provided by the Experimental Animal Center of Southern Medical University and certified by Guangdong Provincial Science Bureau. HCT116 cells stably overexpressing CSRP2 lentivirus (2 × 10^6^) and control cells (6 mice in each group) or CSRP2 stably knockdown SW480 cells and control cells (6 mice in each group) were injected subcutaneously into the left and right hind legs of mice. Use a digital caliper to estimate the tumor volume (volume = (length × width^2^) /2) from two vertical axes every four days, and take a general picture of the tumor. The primary tumor was surgically resected, dehydrated, fixed, embedded in paraffin, and sectioned. The sections were stained with hematoxylin-eosin and observed under the microscope.

### Mouse model of orthotopic liver metastasis

As mentioned earlier, a mouse model of orthotropic metastasis of colorectal cancer was established 14, 15. Balb/C-nu/nu nude mice (3-4 weeks old) (Animal Center, Southern Medical University, Guangzhou, China) were anesthetized and disinfected. After laparotomy, the cells (2 × 10^6^ / mouse) were injected into the subserous layer of cecum in nude mice. The mice were killed on the 60nd day after surgery, and the intestinal and liver tissues were separated, and the metastasis was observed by histological analysis.

### *In vivo* imaging of firefly Luc activity

A scheme of non-invasive detection of luciferase activity in nude mice expressing Luc by bioluminescence using an instrument (FX Pro, USA) 16.

### P130Cas and Rac1 rescue experiments

p130Cas-WT plasmid, p130 Cas-F15 and Rac1 activation inhibitors (NSC23766, Selleck Chemicals, Houston, TX, USA) were added to cells SW480/shCSRP2, HCT15/shCSRP2 and its control cells respectively. WB analysis of p130Cas phosphorylation (20 minutes after culture) and Biochem Kit analysis of Rac1 activation test (24 hours after culture) was performed. The cells were then harvested for the same cell function test as before.

### Ethics approval and consent to participate

All experiments performed in this study are following the ethical standards of Southern Medical University (Guangzhou, China) and the Declaration of Helsinki. Informed consent does not apply to all data that were going to be analyzed anonymously. All animal experiments were approved by the Animal Care and Use Committee of Southern Medical University and complied with the guidelines for the ethical treatment of animals.

### Statistical analysis

All data were analyzed by SPSS19.0 statistical software package. qPCR was analyzed by *t*-test and one-way ANOVA. The comparison between groups was statistically significant by a 2-tailed paired Student *t*-test. Pearsonʼs chi-squared (χ^2^) test was used to analyze the correlation between the expression of CSRP2 and clinical-pathological factors. For patients with different levels of CSRP2 expression, the Kaplan-Meier method was used to draw the survival curve. It is considered that *P* < 0.05 is statistically significant.

## Results

### CSRP2 is downregulated in CRC and associated with advanced tumor progression and poor prognosis of CRC

**Figure 1:**
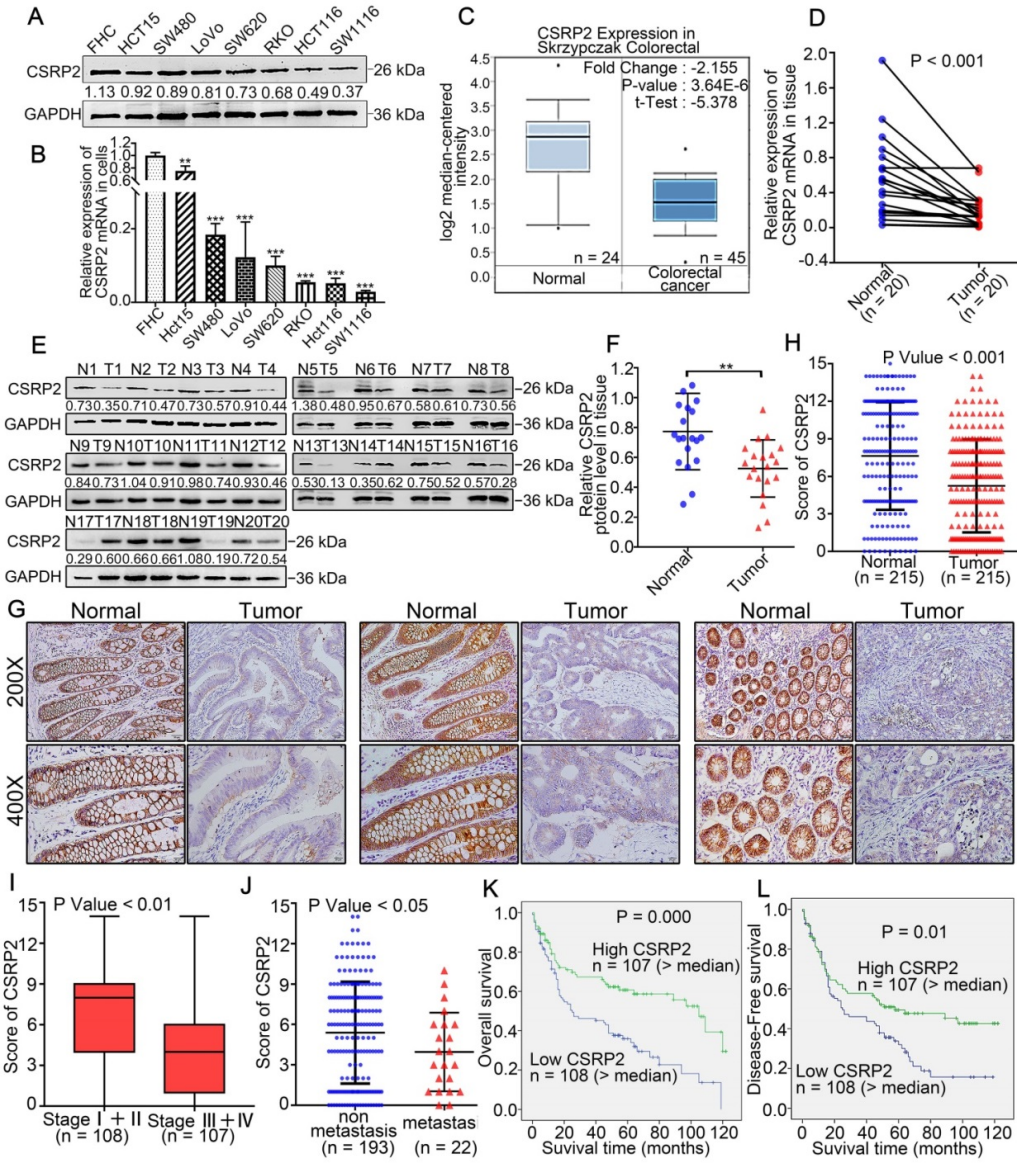
** CSRP2 is downregulated in CRC and correlated with advanced progression and poor prognosis of CRC.** (**A**) Expression of CSRP2 protein was detected in normal colon cell line FHC and seven different colorectal cancer cell lines. (**B**) Expression of CSRP2 mRNA was detected in FHC and seven different colorectal cancer cell lines. Data are shown as mean ± SEM. (**C**) The CSRP2 mRNA expression profile in Skrzypczak Colorectal database. (**D**) The relative CSRP2 mRNA was detected in 20 pairs of fresh CRC tissues and matched paracancerous tissues (*P <* 0.001). (**E**) Expression of CSRP2 protein was detected in 20 paired fresh CRC tissues and matched paracancerous tissues. (**F**) Scatter diagram represents relative CSRP2 protein expression in CRC tissues and paired paracancerous tissues (*P <* 0.01). (**G**) Representative immunohistochemical images of CSRP2 staining in well, moderately and poorly differentiated paraffin-embedded CRC tissues and their adjacent normal mucosa. The scale represents 50 µm. (**H**) IHC staining score of 215 CRC cases and adjacent normal mucosa (*P <* 0.001). (**I**) IHC staining score of CRC cases respectively belonging to Stage I+II and Stage III+IV (*P <* 0.01). (**J**) IHC staining score of non-metastatic and metastatic paraffin-embedded CRC tissues of 215 cases (*P <* 0.05). (**K**) Kaplan-Meier survival analysis showed that the overall survival rate of the low CSRP2 expression group were lower than those of the high expression group (*P =* 0.000). (**L**) Kaplan-Meier survival analysis showed that the disease-free survival rate of the low CSRP2 expression group were lower than those of the high expression group (*P <* 0.01).

qPCR and WB analysis showed that the expression level of CSRP2 in CRC cell lines, including SW480, HCT15, SW620, LoVo, RKO, HCT116, and SW1116, was down-regulated compared with a normal colon cell line (FHC) (Figure 1A-B). We employed the oncogene chip database and web-based data mining platform Oncomine to identify the expression level of CSRP2 in CRC tissues. The results showed that CSRP2 was significantly down-regulated in CRC tissues compared with adjacent normal tissues (*P* < 0.001) (Figure 1C). To validate this observation, we used qPCR and WB to analyze the expression of CSRP2 in 20 pairs of fresh CRC tissues and matched adjacent normal mucosa. The expression level of CSRP2 mRNA and protein in CRC tissues was significantly lower than normal tissues (*P* < 0.01) (Figure 1D-F). We further used immunohistochemistry (IHC) to analyze the expression level of CSRP2 in 215 paraffin-embedded CRC tissue samples collected from Nanfang Hospital. CSRP2 was mainly expressed in the cytoplasm and cell membrane of CRC cells (Figure 1G). Compared with adjacent normal tissues, a significantly decreased expression of CSRP2 was observed in 67.4% (145/215) of CRC tumors (Figure 1G). The total score of CSRP2 expression in CRC was significantly lower than that in adjacent normal mucosa (*P* < 0.001) (Figure 1H).

The clinical data of the patients were analyzed to investigate the correlation between the expression of CSRP2 and the clinicopathological features of CRC. The results showed that the expression of CSRP2 protein was negatively correlated with lymph node status (*P* < 0.001), distant metastasis (*P* < 0.05) (Figure 1J), vascular invasion (*P* < 0.001), and clinical stage (*P* < 0.001) (Figure 1I). There was no significant difference between its expression and age, sex, tumor size, tumor location, and differentiation (*P* > 0.05, Supplementary Table S1 and Table S2). By analyzing the tumor size in patients with clinical stage Ⅰ+ Ⅱ and stage Ⅲ + Ⅳ, we found that CSRP2 expression in stage Ⅰ + Ⅱ, but not in stage Ⅲ + Ⅳ, was related to tumor size (Supplementary Table S3). Also, the Kaplan-Meier survival analysis showed that the overall survival rate and disease-free survival rate of the low CSRP2 expression group were lower than those of the high expression group (*P* < 0.001, *P* < 0.01, Figure 1K-L).

### Overexpression of CSRP2 inhibits aggressive phenotype and tumorigenicity of CRC cells

**Figure 2:**
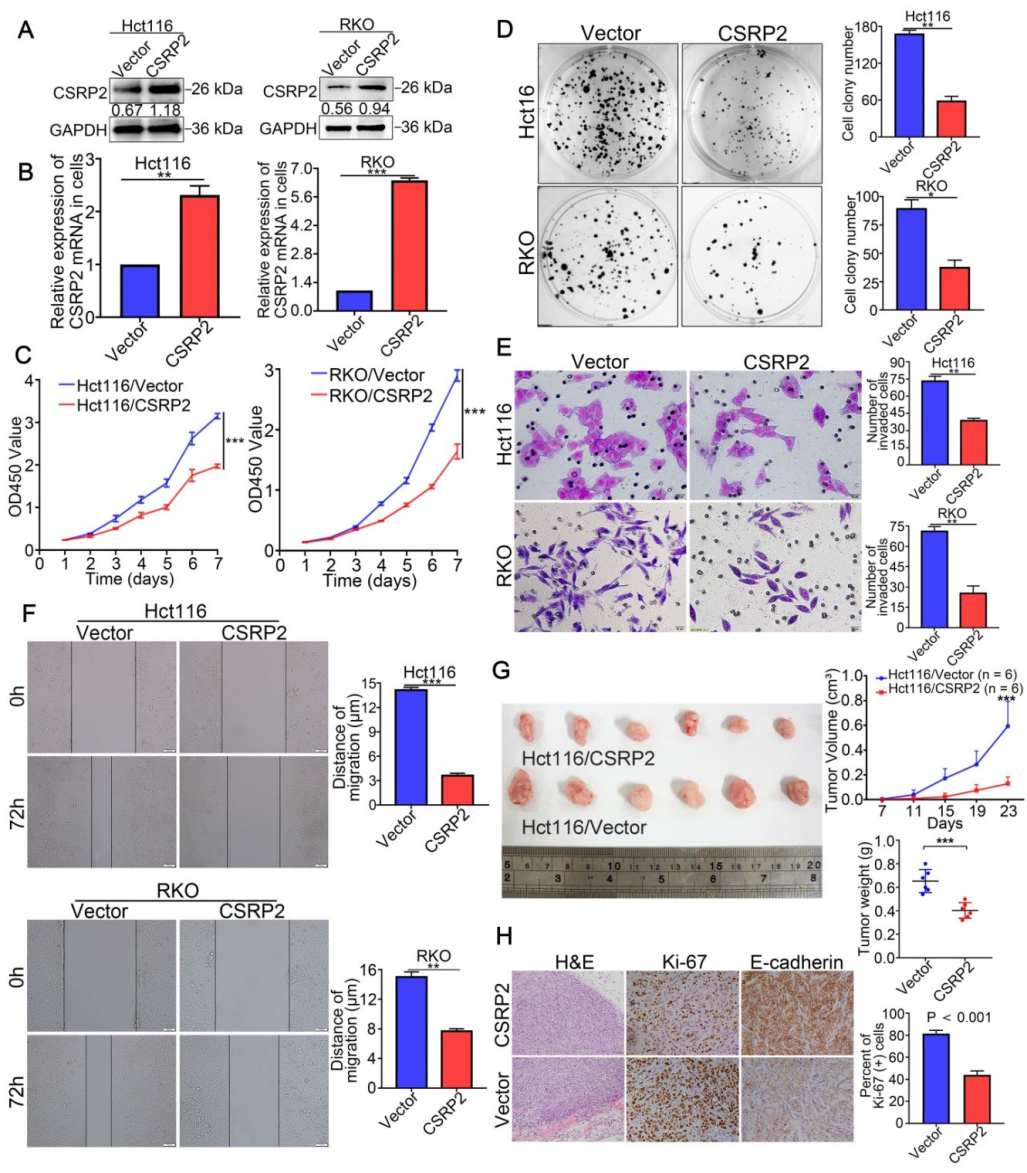
** CSRP2 suppresses CRC cell proliferation, migration and invasion.** (**A**) Overexpression of CSRP2 was confirmed in HCT116 and RKO cells by WB. (**B**) Overexpression of CSRP2 was confirmed in HCT116 and RKO cells by qPCR. (**C**) CCK8 assay assessed the proliferation ability of CSRP2-overexpressed CRC cells. (**D**) Colony formation assay assessed the proliferation ability of CSRP2-overexpressed CRC cells. (**E**) Transwell invasion assay assessed invasive ability of CSRP2-overexpressed CRC cells. (**F**) Wound healing assay assessed migration capability of CSRP2-overexpressed CRC cells. (**G**) The determination of subcutaneous tumor of nude mice respectively constructed by CSRP2-overexpressed CRC cells and control cells. (**H**) H&E, Ki-67 and E-cadherin staining of subcutaneous tumor. Data are shown as mean ± SEM.

To study the possible function of CSRP2 in the progression of CRC, two CRC cell lines with stable overexpression of CSRP2, HCT116/CSRP2, and RKO/CSRP2, were established. HCT116 and RKO transfected with an empty lentiviral vector were used as the negative controls. WB and qPCR analysis confirmed that the transfection was effective (Figure 2A-B). CCK8 and colony formation assays showed that CSRP2 overexpression inhibited proliferation of HCT116 and RKO cells (Figure 2C-D). Also, overexpression of CSRP2 significantly inhibited the migration and invasion of CRC cells detected by wound healing and Transwell invasion assays (*P* < 0.05, Figure 2E-F). Furthermore, the effect of CSRP2 on tumor growth *in vivo* was analyzed following subcutaneous injection of HCT116/CSRP2 cells and control cells in nude mice. As shown in Figure 2G, the tumors derived from HCT116/CSRP2 cells developed slower than the negative control with a smaller tumor volume (Figure 2G; n = 6; P < 0.05). In addition, IHC staining confirmed that the Ki-67 proliferation index of tumors derived from HCT116/CSRP2 cells was lower than from HCT116/Vector cells (Figure 2H; P < 0.001).

### Down-regulation of CSRP2 promotes aggressive phenotypes and tumorigenicity of CRC cells

**Figure 3:**
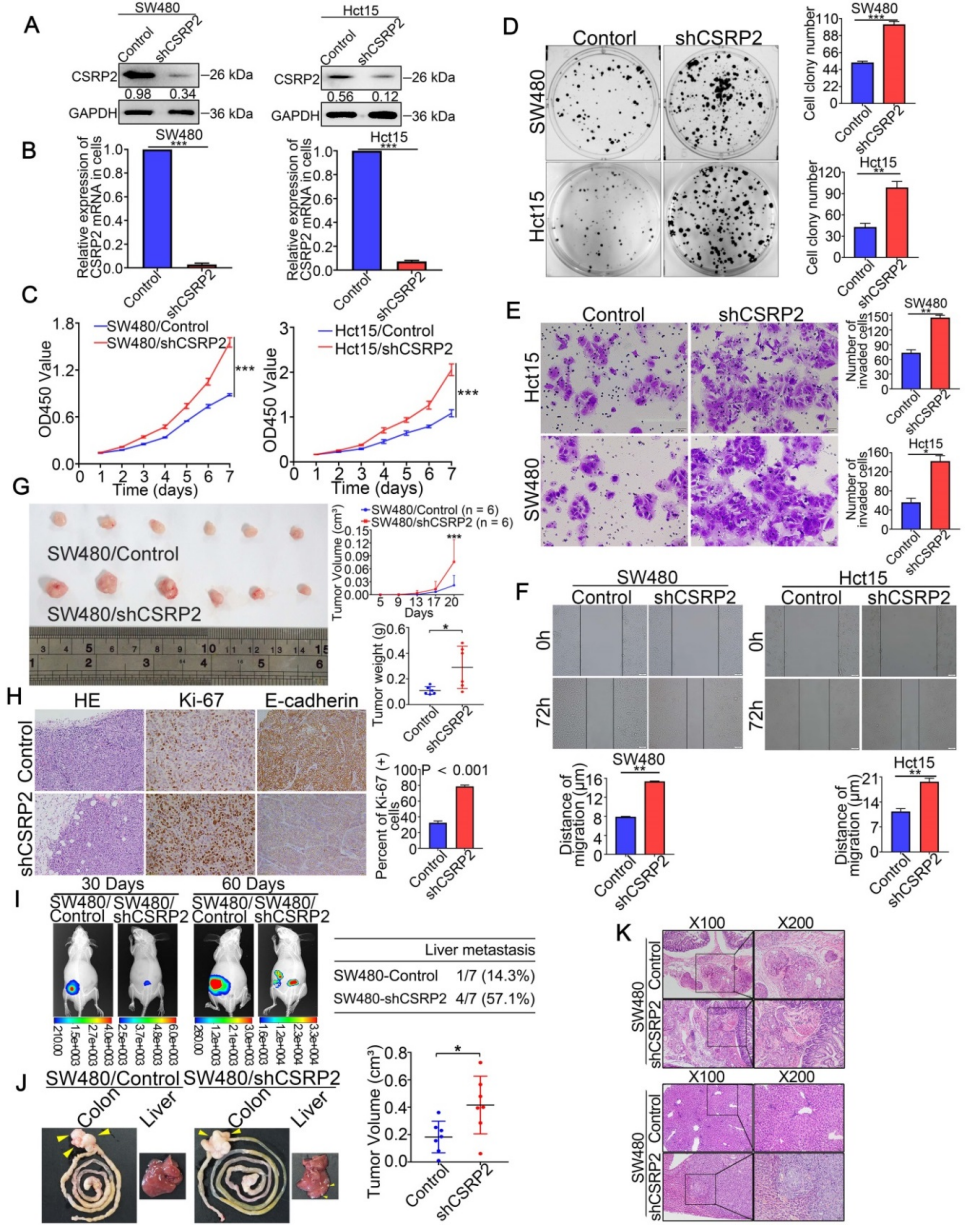
** CSRP2 depletion promotes the progression of CRC cells *in vitro* and *in vivo*.** (**A**) Western blot analysed confirmed the successful construction of stable CSRP2 depletion in SW480 and HCT15 cells. (**B**) qPCR analysed confirmed the successful construction of stable CSRP2-depletion in CRC cells. (**C**) CCK8 assay assessed the proliferation ability of CSRP2-depleted CRC cells. (**D**) Colony formation assay assessed the proliferation ability of CSRP2-depleted CRC cells. (**E**) Transwell invasion assay assessed invasive ability of CSRP2-depleted CRC cells. (**F**) Wound healing assay showed migration capability of CSRP2-depleted CRC cells. (**G**) The determination of subcutaneous tumor of nude mice respectively constructed by CSRP2-depleted CRC cells and control cells. (**H**) H&E, Ki-67 and E-cadherin staining of subcutaneous tumor. (**I**) Representative photographs of liver metastasis of CSRP2-depleted SW480 cells and control cells on 30 and 60 days post-inoculation by *in vivo* fluorescence imaging (left). Summary results of nude mice presenting with liver metastasis between CSRP2-depleted SW480 group and control group (right). (**J**) Dissection of intestinal and liver in *in situ* implant CRC nude mice. (**K**) The intestinal and liver tissues were stained with H&E and observed under the light microscope. Data are shown as mean ± SEM.

To clarify the effect of CSRP2 knockdown on CRC cells, lentiviral vectors carrying s0pecifically targeted CSRP2 were used to silence the expression of endogenous CSRP2 in SW480 and HCT15 cells (Figure 3A-B). As shown in Figure 3C-3F, the knockdown of CSRP2 promoted the ability of cell proliferation, migration, and invasion. The subcutaneous tumor model showed that the tumors derived from SW480/shCSRP2 cells developed slower than those derived from SW480/Control with a larger tumor volume (Figure 3G; n = 6; P < 0.05). Moreover, the Ki-67 proliferation index of tumors derived from SW480/shCSRP2 cells was higher than those derived from SW480/Control cells (Figure 3H; *P* < 0.001).

### CSRP2 suppresses tumorigenicity and metastasis of CRC *in vivo*

To verify the effect of endogenous CSRP2 on the metastatic ability of CRC, we injected SW480/Control cells and SW480/shCSRP2 cells into the subserosa of the cecum in nude mice to establish the orthotopic liver metastasis model. Luciferin substrate was injected into the abdominal cavity of nude mice on day 30 and 60. Subsequently, luciferase-labeled SW480/shCSRP2 cells were detected by bioluminescence *in vivo* imaging. We found that all 7 pairs of metastases were successfully modeled in mice. The bioluminescence signal of the SW480/shCSRP2 group was stronger than that of the control group with liver metastasis in 4 mice (57.1%) compared to only 1 mouse (14.3%) in the control group (Figure 3I). The nude mice were killed 60 days later and the intestinal and liver tissues of the nude mice were dissected. The size of colon tumors in the SW480/shCSRP2 group was significantly larger than the control group (Figure 3J; *P* < 0.05). The intestinal and liver tissues were stained with H&E and the metastasis was confirmed by microscopy (Figure 3K).

### CSRP2 inhibits EMT and modulates phosphorylation of p130Cas and activation of Rac1 in CRC cells

**Figure 4:**
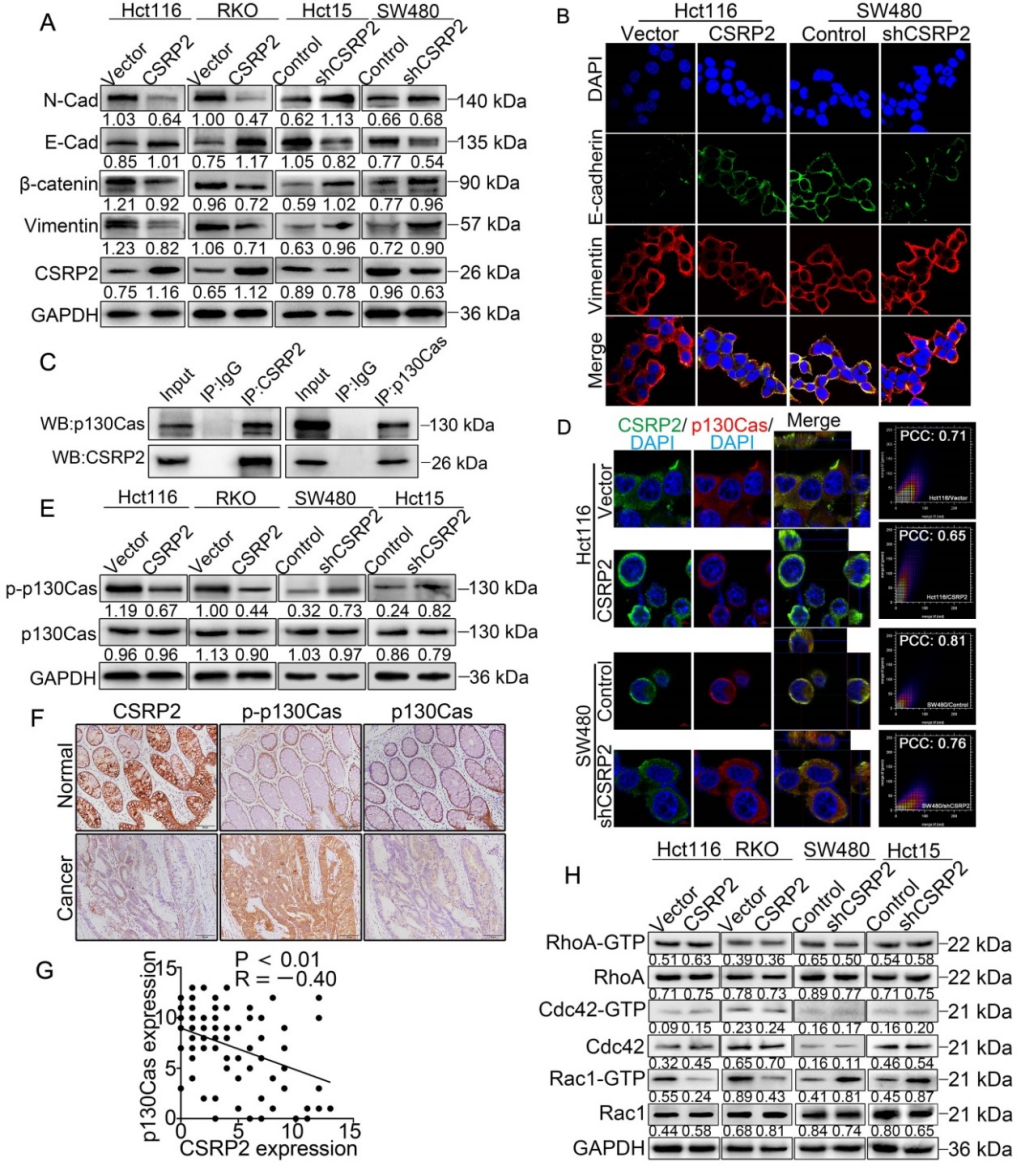
** CSRP2 inhibits EMT and modulates p130Cas phosphorylation and Rac1 activation in CRC cells.** (**A**) Western blot was performed to detect the EMT marker proteins expression alteration of CSRP2-overexpressed or -depleted cells compared to control cells. (**B**) IF staining analyzed the EMT marker proteins expression alteration of CSRP2-overexpressed or -depleted cells compared to control cells. (**C**) Interaction between CSRP2 and p130Cas was confirmed by co-immunoprecipitation assay. (**D**) IF staining assay with Z-stack was used to confirme the interaction between CSRP2 and p130Cas. Using co-location scatter diagram and PCC to analyze co-location (PCC: Pearson correlation coefficient). (**E**) Western blot was performed to detect the protein expression changes of p130Cas and phosphorylated p130Cas (p-p130Cas) between CSRP2-overexpressed or -depleted cells and control cells. (**F**) Immunohistochemical detection of the correlation between CSRP2 and p-p130Cas in serial sections of the same CRC tissues and adjacent normal mucosa. (**G**) The chart is the statistics of correlation coefficient of figure F. (**H**) Protein expression alterations of Rac1, RhoA, Cdc42, and their active form, were detected by western blot in CSRP2-overexpressed or-depleted cells and control cells.

Liver metastasis is considered the leading cause of death in patients with CRC. Studies have shown that abnormal EMT-related markers are an important cause of tumor cell growth, migration, and invasion 17. WB showed that with an increase in the CSRP2 protein, the expression of epithelial marker E-cadherin was up-regulated, while that of N-cadherin, β-catenin, and vimentin was down-regulated. Opposite results were observed with the inhibition of CSRP2 (Figure 4A). Immunofluorescence (IF) analysis further confirmed that overexpression of CSRP2 inhibited EMT in CRC cells (Figure 4B; Supplementary Figure S1A-B). More significantly, CSRP2 could inhibit the invasion and migration of CRC cells *in vitro* (Figure 2E-F and Figure 3E-F) and liver metastases of tumors *in vivo* (Figure 3I and K). These results implied that CSRP2 might impede the invasion and metastasis of CRC cells by inhibiting EMT.

Next, we explored the mechanism of CSRP2 inhibition of EMT involved in the progression of CRC. An interaction between CSRP2 and p130Cas in mouse vascular smooth muscle was previously reported 18. Studies have also reported an abnormal activation of the p130Cas/BCAR1 signaling network in various cancers that may lead to the upregulation of regulatory pathways, which can promote cell transformation 19. We, therefore, explored whether CSRP2 interacts with p130Cas in CRC cells and whether CSRP2 participates in the progression of CRC by affecting the expression and/or phosphorylation activity of p130Cas. We detected an interaction between CSRP2 and p130Cas by co-immunoprecipitation (Co-IP) (Figure 4C). The IF assay further confirmed the co-localization of CSRP2 and p130Cas (Figure 4D; Supplementary Figure S1B). WB and IF assay showed no significant change in total p130Cas in CRC cells by either overexpressing or silencing CSRP2 (Figure 4D-E; Supplementary Figure S1C). However, p130Cas often functions *via* phosphorylation. There are three most common tyrosine kinase sites, Tyr165, Tyr249, and Tyr410, in the substrate domain, which play important roles in cytoskeleton remodeling, migration, and invasion 20, 21. We used antibodies of the phosphorylated Tyr410 site to detect the phosphorylation level of p130Cas and found that the phosphorylated p130Cas (p-p130Cas) was down-regulated in CRC cells overexpressing CSRP2, while the p-p130Cas was up-regulated in CRC cells lacking in CSRP2 (Figure 4E; Supplementary Figure S1D-E). Also, the phosphorylation level of p130Cas in the samples with low expression of CSRP2 was increased, as detected by IHC using serial sections of tumors. This result was confirmed by WB with proteins extracted from fresh CRC tissues. Furthermore, E-cadherin was down-regulated and vimentin was up-regulated in fresh CRC tissues with low expression of CSRP2 (Figure 4F-G; Supplementary Figure S2A).

It has been reported that phosphorylation of p130Cas leads to the activation of the Rho GTPase Rac1 22. In many cancers, such as medulloblastoma, breast, ovarian, and various other cancers, p130Cas activates Rac1 and affects cancer progression 23-25. We speculated that the up- or down-regulation of CSRP2 would activate Rac1 in CRC. Rac1 activation kit was used to monitor the activation of Rac1, and the results showed that the activated form of Rac1 (Rac1-GTP) was decreased following overexpression of CSRP2, while it was increased after silencing CSRP2 (Figure 4H). There was no significant difference in the activation of two other Rho GTPases, RhoA and Cdc42, between CSRP2 overexpression or its silencing in CRC and control cell lines (Figure 4H). Since p130Cas phosphorylation is often activated by Src, dasatinib, a Src inhibitor 26, was added to CSRP2-silenced cell lines, SW480/shCSRP2 and HCT15/CSRP2, and their control groups. The results showed that dasatinib could reverse the phosphorylation level enhanced by p130Cas after silencing CSRP2 and also reversed the enhanced invasive ability of cells (Supplementary Figure S2B-C).

### CSRP2 suppresses EMT through the Hippo, ERK, and PAK signaling pathways in CRC

**Figure 5:**
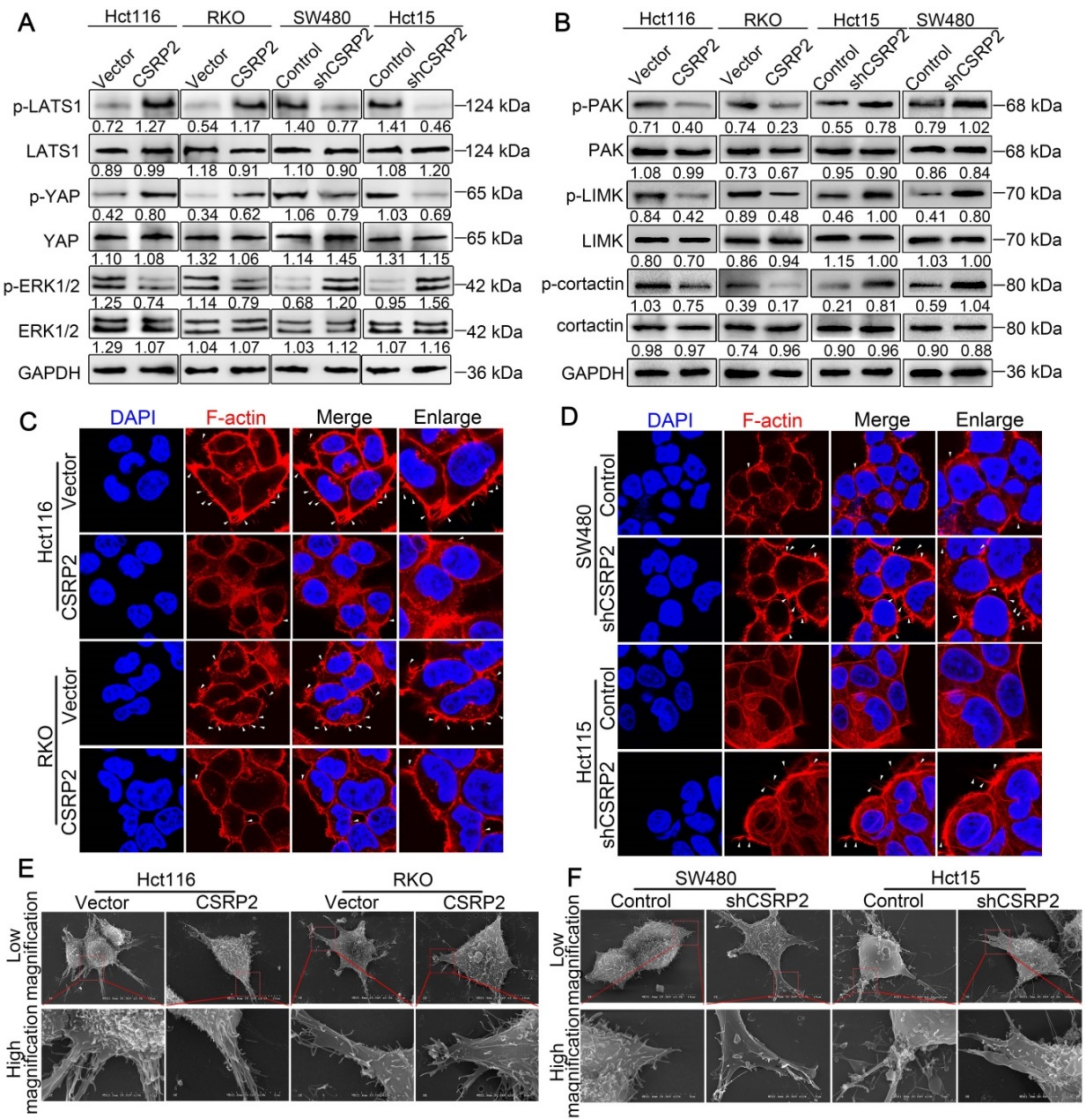
** CSRP2 affectes the Hippo, ERK, and PAK signaling pathways and the pseudopodia formation in CRC cells.** (**A**) WB was performed to detect the activation of Hippo and ERK pathways in CSRP2-overexpressed or -depleted CRC cells and control cells. (**B**) WB was performed to detect the activation of PAK, LIMK and cortactin in CSRP2-overexpressed or -depleted CRC cells and control cells. (**C**) IF staining analyzes the pseudopodia of CSRP2-overexpression CRC cells. (**D**) Phalloidin IF staining analyzes the pseudopodia of CSRP2-depletion CRC cells. (**E**) The pseudopodia of CSRP2-overexpression CRC cells was detected by scanning electron microscope; (**F**) The pseudopodia of CSRP2 depletion cells was detected by SEM.

Previous studies have shown that RhoA/Rac1 GTPase is an important link with Hippo, ERK, and PAK signaling pathways involved in tumor progression 27-34. The Hippo signaling pathway is an evolutionarily conserved kinase cascade pathway, a key regulator of development and homeostasis in tissues, and its dysregulation contributes to cancer development 32, 33. We hypothesized that CSRP2 affects the development of CRC by activating the Hippo pathway. The WB analysis showed no significant change in total LATS1 and YAP proteins following overexpression of CSRP2, while phosphorylated LATS1 and YAP were up-regulated. The opposite result was obtained after silencing CSRP2 (Figure 5A).

Several recent studies have shown that activation of Rac1 could promote EMT and tumor progression by activating the ERK signaling pathway 31, 34. Rac1 also influenced pseudopodia changes through the PAK-LIMK signaling pathway, leading to cell migration, invasion, and EMT 27, 28, 35. We explored whether CSRP2 affected the PAK-LIMK and ERK signaling pathways to regulate EMT and metastasis of CRC. WB showed that CSRP2 did not affect the expression of ERK1/2, PAK, LIMK, and cortactin, but down-regulated the phosphorylated forms of these proteins (Figure 5B). The PAK-LIMK-cortactin signaling pathway was reported to mainly promote cell pseudopodia 27, 36-38. Our IF results showed that the size of plasma membrane bubbles of shCSRP2 cells decreased significantly, and the pseudopodia (microspikes) became weaker, shorter, and sparser than the control group (Figure 5C-D). SEM also found that shCSRP2 cells had significantly elongated and enlarged pseudopodia, while the control cells had only a few short pseudopodia (Figure 5E-F). Collectively, these data suggested that CSRP2 might suppress EMT through the Hippo, ERK, and PAK signaling pathways in CRC.

### CSRP2 suppresses CRC progression *via* p130Cas/Rac1-modulated Hippo, ERK, and PAK signaling pathways

**Figure 6:**
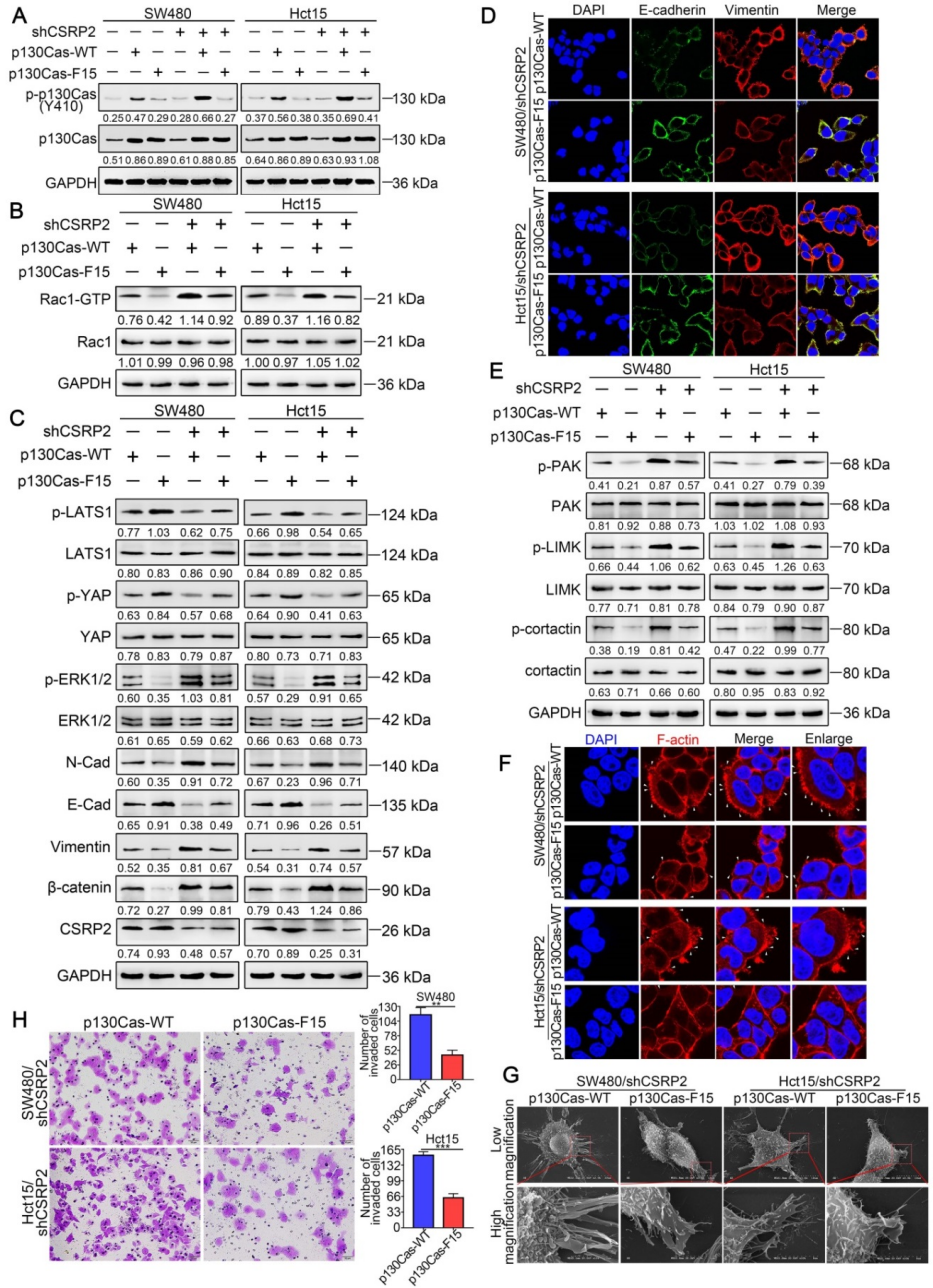
** CSRP2 regulates Hippo, ERK, and PAK signaling pathways through p130cas/Rac1 mediation to inhibit CRC cell invasion and EMT.** (**A**) WB was performed to deteced the expression of total p130Cas and p-p130Cas in CSRP2-depleted CRC cells and control cells treated with wild-type and mutant p130Cas. (**B**) Rac1 activation was detected by WB. (**C**) WB was performed to detect the activation of LATS1, YAP in Hippo signal pathways and the activation of ERK in ERK signal pathways, and the protein expression changes of EMT markers. (**D**) The expression of E-cadherin and vimentin were analyzed by IF. (**E**) WB was performed to detect the activation of PAK, LIMK and cortactin in the skeleton signal pathway. (**F**) Phalloidin IF staining analyzes the pseudopodia. (**G**) The changes of pseudopodia were detected by SEM. (**H**) Transwell invasion assay was performed to analyze the invasive ability of cells.

To further clarify the above mechanism, we used wild-type p130Cas (p130Cas-WT) and mutant p130Cas (p130Cas-F15) to transfect SW480/shCSRP2, HCT15/shCSRP2 and control cells, 39, and detected a variety of changes in many relevant proteins. We found that the phosphorylation level of p130Cas in p130Cas-F15- transfected SW480/shCSRP2 and HCT15/shCSRP2 cells was significantly decreased (Figure 6A) while there was no change in the decreased CSRP2 expression (Figure 6C). Also, the elevated Rac1-GTP was decreased (Figure 6B) but the total protein levels of LATS1 and YAP showed no significant difference, albeit their corresponding phosphorylation levels were increased. Finally, the total protein level of ERK1/2 remained unchanged but the up-regulated p-ERK1/2 level was decreased (Figure 6C). Meanwhile, EMT epithelial marker E-cadherin was up-regulated, while interstitial markers N-cadherin, β-catenin, and vimentin were down-regulated (Figure 6C). IF analysis also showed that p130Cas-F15 inhibited the EMT in CRC cells (Figure 6D; Supplementary Figure S2D). The total proteins of PAK, LIMK and cortactin in the PAK signaling pathway were not changed significantly, but the corresponding phosphorylation levels were decreased (Figure 6E). The pseudopodia formation in p130Cas-F15 cells was weaker, shorter, and sparser than in the p130Cas-WT cells, as is evident from the IF analysis (Figure 6F). The SEM results were similar to those of IF (Figure 6G). Transwell invasion assay showed that the invasive ability of SW480/shCSRP2 and HCT15/shCSRP2 cells treated with p130Cas-F15 was significantly weaker than that in the p130Cas-WT group (Figure 6H).

**Figure 7:**
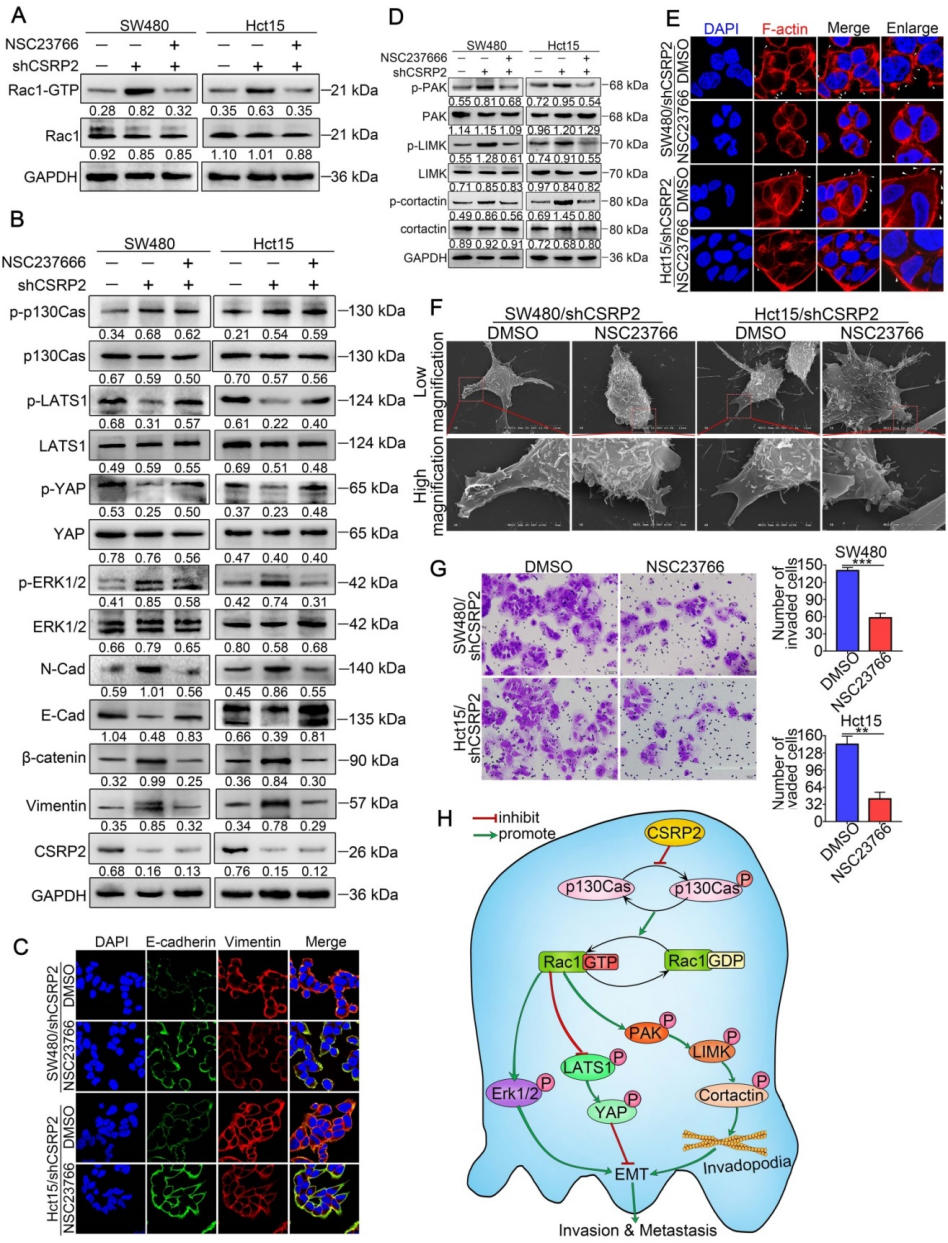
** CSRP2 regulates Hippo, ERK, and PAK signaling pathways to suppresses CRC cell invasion and EMT *via* Rac1.** (**A**) Rac1 activation was detected by WB in CSRP2- depleted CRC cells and control cells treated with Rac1 inhibitor NSC23766. (**B**) WB was performed to detect the activation of LATS1, YAP in Hippo signal pathways and the activation of ERK in ERK signal pathways, and the changes of EMT markers. (**C**) The expression of E-cadherin and vimentin were analyzed by IF. (**D**) WB was performed to detect the activation of PAK, LIMK and cortactin in the skeleton signal pathway. (**E**) Phalloidin IF staining analyzes the pseudopodia. (**F**) The changes of pseudopodia were detected by scanning electron microscope. (**G**) Transwell invasion assay was used to analyze the changes of invasive ability of cells; (**H**) Schematic diagram showing the mechanism of action of CSRP2 in CRC. CSRP2 could inhibit the activation of Rac1 by inhibiting the p-p130cas, thus promoting the activation of the Hippo pathway, and simultaneously inhibiting the PAK-LIMK-cortactin and ERK signal pathway, thereby inhibiting the EMT and metastasis of CRC.

Furthermore, we treated SW480/shCSRP2 and HCT15/shCSRP2 cells with NSC23766, an inhibitor of Rac1 activation 40. The results showed that NSC23766 in CSRP2 significantly reduced the level of Rac1-GTP in CSRP2-downregulated CRC cells (Figure 7A), but there was no significant change in CSRP2, p130Cas, and p-p130Cas. There was also no significant difference in the total protein levels of LATS1 and YAP, but the corresponding phosphorylation levels were increased. On the other hand, although the total protein level of ERK1/2 remained unchanged, the level of up-regulated p-ERK1/2 decreased (Figure 7B). The EMT epithelial marker E-cadherin was up-regulated, while interstitial markers N-cadherin, β-catenin, and vimentin were downregulated (Figure 7B). IF assay also showed that E-cadherin, an EMT epithelial marker, was up-regulated, while vimentin, a stromal marker, was downregulated (Figure 7C). The total proteins of PAK, LIMK, and cortactin in the PAK signaling pathway had no significant change, but the corresponding phosphorylation levels decreased (Figure 7D). Both IF and SEM analyses showed that the pseudopodia of cells treated with NSC23766 were decreased (Figure 7E & 7F; Supplementary Figure S2E). The invasion ability of SW480/shCSRP2 and HCT15/shCSRP2 cells treated with NSC23766 was significantly weaker than that of control cells, as was evident by the Transwell invasion assay (Figure 7G). These observations confirmed that CSRP2 could suppress CRC progression *via* p130Cas/Rac1-modulated Hippo, ERK, and PAK signaling pathways.

## Discussion

As a major cause of death in CRC patients, metastasis has attracted much attention in CRC research. However, the molecular mechanisms underlying CRC metastasis remain largely obscure. In this study, we first detected the role of CSRP2 in the development of CRC and studied the molecular mechanisms by which CSRP2 regulates CRC metastasis.

CSRP2 belongs to the three-member protein (CSRP1-3) CSRP family, which contains two functional Lim domains linked to amino acid-rich repeats that are closely related to their function. CSRPs are associated with the cytoskeletal regulation by interacting with the actin cross-linking protein, α-actin, the adhesion plaque protein zyxin, and the scaffold protein p130Cas 18, 41-43. Co-sedimentation and ELISA assays suggested a direct association of CSRP2 with F-actin 44, 45. Furthermore, CSRP2 could also sequester the scaffold protein p130Cas at focal adhesions, control lamellipodia formation, and reduce vascular smooth muscle cell motility 18, 46. These observations suggested that CSRP2 may be involved in the cytoskeletal regulation and cell motility, processes that are associated with cancer metastasis.

Although there is limited information on the role of CSRP2 in cancers, few studies suggested that it plays an important role in some cancers, such as gastric cancer, HCC and breast cancer 9-11. Intriguingly, CSRP2 appears to have different functions in these three cancers. CSRP2 is thought to inhibit the invasion and metastasis of cancer cells in gastric cancer and may be related to the progression and dedifferentiation of HCC, but has an opposite role in breast cancer. Therefore, the underlying mechanisms of CSRP2 function in various cancers need to be carefully examined 9, 10. So far, the role and molecular mechanism of CSRP2 in CRC remain unknown. Using various methods, including bioinformatics analysis, biochemical experiments, and statistical analysis, we found that CSRP2 is significantly down-regulated in CRC, and its low expression is associated with metastasis and poor prognosis in patients with CRC. To further study the biological function of CSRP2 in CRC, we performed gain- and loss-of-function experiments in CRC cells. Our results revealed that CSRP2 could inhibit the proliferation, migration, invasion, and tumorigenicity of CRC cells. These findings suggested that CSRP2 may be a tumor suppressor gene in the development and progression of CRC.

EMT is a key mechanism involved in the complex process of tumor invasion and metastasis. During cancer progression, EMT can alter adhesion of epithelial cancer cells that invade the surrounding tissues and migrate to distant sites, thus contributing to tumor metastasis. Our data showed that overexpression of CSRP2 increased the level of E-cadherin and decreased that of vimentin, β-catenin, and N-cadherin in CRC cells, while silencing of CSRP2 induced opposite results. These data demonstrated that CSRP2 could inhibit EMT in CRC cells by suppressing the invasion and migration of CRC cells.

Mechanistically, we analyzed the regulation of CSRP2/p130Cas/Rac1 regulatory network. P130Cas is a universal scaffold molecule that contains several structural motifs, including a Src homology 3 (SH3) domain, a proline-rich domain, a substrate domain with 15 YxxP repeats (YxxP 15), and a C-terminal homologous domain of the Cas family. The main post-translational modification of p130Cas is the phosphorylation of tyrosine and serine/threonine residues at the three most common tyrosine kinase sites, Tyr165, Tyr249, and Tyr410. Accumulating evidence has revealed that the phosphorylation of p130Cas and its interactions with other proteins can regulate the cytoskeletal rearrangement, enhance cell migration and invasion, promote tumorigenesis and EMT, and thus play a crucial role in tumor progression 19, 47. Recent studies have revealed that the phosphorylation of p130Cas is closely related to Rac1 activation 48, 49 and can promote actin polymerization and recruitment of high-affinity integrin receptors necessary for the expansion of lamella and cell migration 30. Rac1 is an important member of the GTPase Rho family, the intracellular coordinator of cell migration signals, and is involved in tumorigenesis, proliferation, invasion, and metastasis through multiple signaling pathways in various cancers 50, 51. Previously, the interaction between CSRP2 and p130Cas has been reported 18. Our results suggested that CSRP2 suppressed Rac1activation by inhibiting the phosphorylation of p130Cas, resulting in the suppression of EMT in CRC. Our study also demonstrated that dasatinib, a chemotherapy drug used in the clinic and an inhibitor of p130Cas, could impede the enhanced phosphorylation of p130Cas after CSRP2 knockdown.

Numerous studies have shown that Rac1 is intricately linked with Hippo, ERK, and PAK signaling pathways in tumor progression 27-34. Rac1 activation is believed to stimulate the PAK/LIMK/cortactin axis and affect the actin cytoskeleton 35, causing changes in cell pseudopodia, an important morphological feature of EMT. Our study showed that CSRP2 could block the activation of PAK/LIMK/cortactin axis by inhibiting Rac1 activation, thus preventing the polymerization of F-actin and formation of long and thick pseudopodia (microspikes). Therefore, we believe that CSRP2 negatively impacts EMT by inhibiting the PAK/LIMK/cortactin axis.

It has been reported that p130Cas is associated with LIM proteins zyxin, TRIP6, and Ajuba, and controls cell migration by regulating downstream signaling events 22, 41. As a LIM protein, zyxin can affect the activation of Hippo 52. The core components of the Hippo pathway are MST1/2, SAV1, LATS1/2 (Wts in Drosophila), and MOB1. Among them, SAV1 and MOB1 are adapter proteins, which can bind to MST1/2 and LATS1/2 respectively and enhance their phosphorylation. YAP is the main effector molecule downstream of the bypass pathway and can be directly phosphorylated by LATS1/2. The Hippo pathway negatively regulates YAP activity through this cascade phosphorylation and then regulates cell proliferation, invasion, and metastasis in cancer 53. Herein, we found that, by promoting the phosphorylation of YAP, CSRP2 could inhibit EMT and invasion in CRC cells through Rac1.

It has previously been shown that Rac1 can regulate the ERK signal to cope with endoplasmic reticulum stress 31. In mammalian cells, the intracellular signal transduction pathways associated with ERK are considered classical MAPK signal transduction pathways, which play an important role in the development of tumors 54. Our data suggested that CSRP2 can inhibit the ERK signaling pathway *via* Rac1, suppressing EMT and invasion in CRC cells. To the best of our knowledge, our study is the first to elucidate the role of CSRP2 in CRC progression and illustrate the CSRP2/p130Cas/Rac1 regulatory network in EMT and metastasis of CRC. Nevertheless, the specific upstream mechanism causing CSRP2 downregulation in CRC and regulation of its downstream targets need to be further illustrated.

## Conclusions

In this study, we detected down-regulation of CSRP2 in most CRC specimens and CRC cell lines associated with the progression and poor prognosis of CRC. CSRP2 suppressed the proliferation, migration, and invasion of CRC cells. Mechanistic studies showed that CSRP2 could inhibit the phosphorylation of p130Cas and activation of Rac1, which then activated the Hippo pathway and inhibited PAK-LIMK-cortactin and ERK signaling pathways, thereby impeding EMT and metastasis of CRC. Thus, our results revealed a novel mechanism of the CSRP2/P130Cas/Rac1 regulatory axis involved in the regulation of CRC metastasis (Figure 7H).

## Supplementary Material

Supplementary figures and tables.

## Abbreviations

CRC: colorectal cancer
CSRP2: cysteine-rich protein 2
COIP: co-immunoprecipitation assay
EMT: epithelial-mesenchymal transition
IHC: immunohistochemical
IF: immunofluorescent assay
qPCR: real-time quantitative PCR
WB: western blot
