# Supplementary material for: CSRP2 suppresses colorectal cancer progression via p130Cas/Rac1 axis-meditated ERK, PAK, and HIPPO signaling pathways
Source: Theranostics. 2020 Sep 2;10(24):11063–79. doi: 10.7150/thno.45674 (PMC7532686; doi:10.7150/thno.45674)
Supplement: Supplementary file 1 — Supplementary figures and tables. [file thnov10p11063s1.pdf]

supplementary figures and supplementary tables:

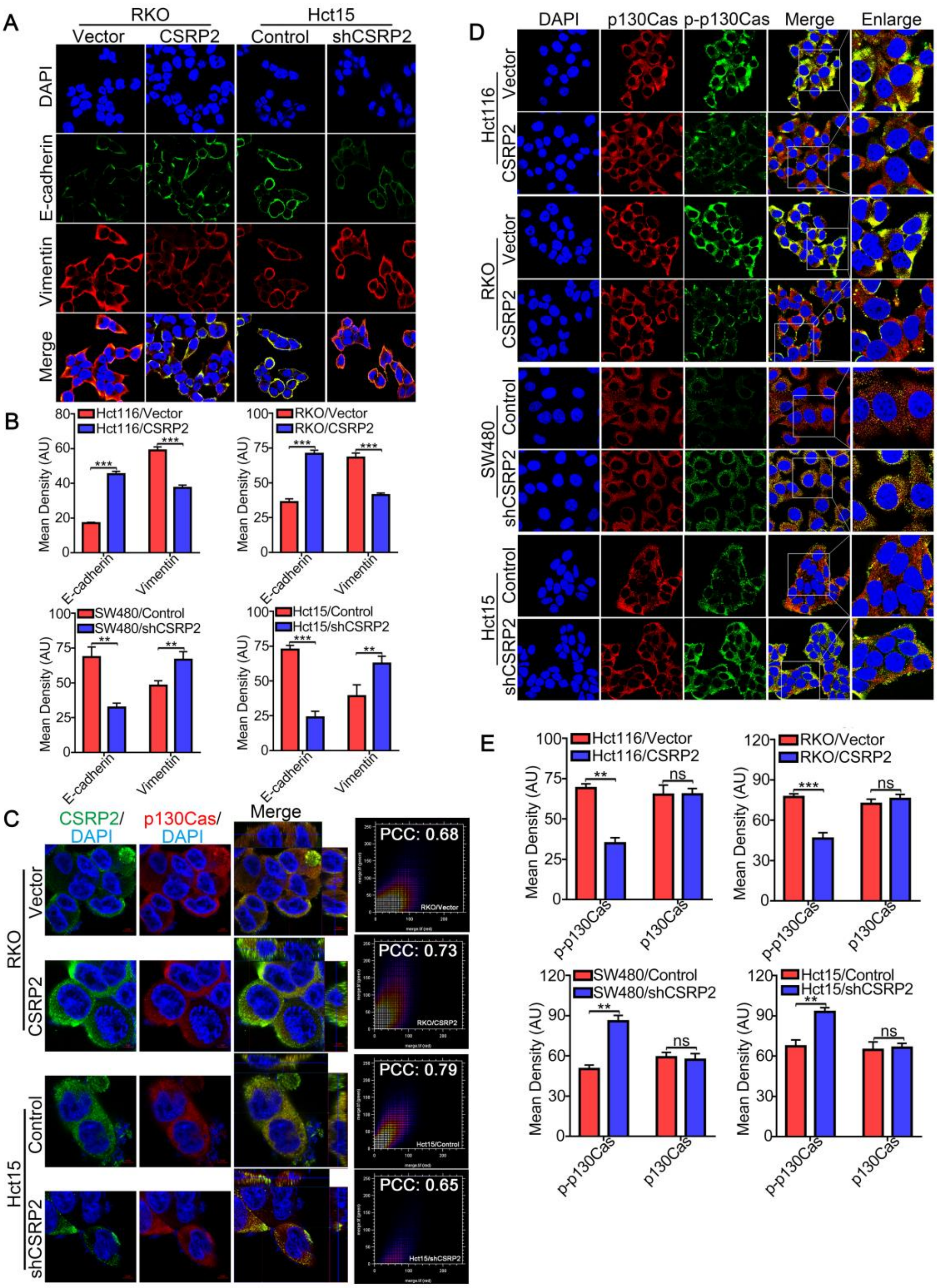

Supplementary Figure S1. (A) IF assay were performed to detect the expression of EMT markers in CSRP2-overexpressed or -depleted CRC cells and control cells. (B) The statistics of IF intensity of figure 4B and figure S1A using image J software. the

unit is Arbitrary Units (AU). (C) Interaction between CSRP2 and p130Cas was confirmed by IF assay with Z-stack. Using co-location scatter diagram and PCC to analyze co-location (PCC: Pearson correlation coefficient). (D) IF assays analyzed the protein expression of p130Cas and p-p130Cas in CSRP2-overexpressed or -depleted CRC cells and control cells. (E) The statistics of IF intensity of figure S1D using image J software, the unit is Arbitrary Units (AU).

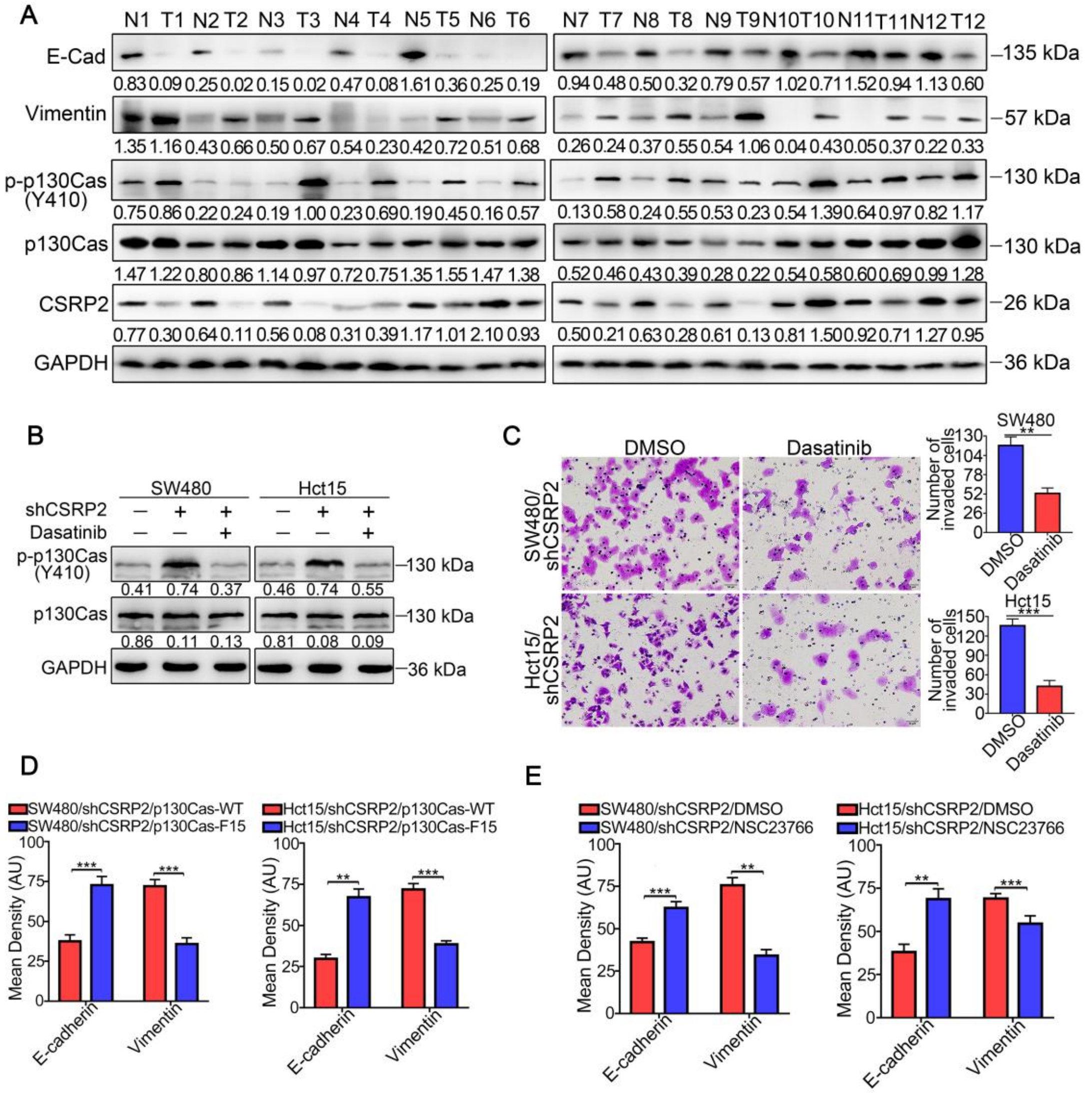

Supplementary Figure S2. (A) WB was performed to detect the protein changes of p130Cas, p-p130Cas and EMT marker in 12 pairs of fresh colorectal cancer tissues. (B) WB was performed to detect the protein changes of p130Cas and p-p130Cas after treating with dasatinib on shCSRP2 cell line. (C) Transwell invasion assay was used to detect the changes of cell invasion ability after treating with dasatinib in shCSRP2 cell line. (D) The Figure shows the statistical analysis of the fluorescence intensity of Figure 6D. (E) The Figure shows the statistical analysis of the fluorescence intensity of Figure 7C. The unit is Arbitrary Units (AU).

Supplementary Table S1. The Relationship between CSRP2 expression and clinic pathological features

Characteristics	CSRP2 expression				
	n=215	Low	High	x2 value	**P** value
Frequency	215	145(67.4%)	70(35.6%)		
**Gender**				0.012	0.514
Male	124	84	40		
Female	91	61	30		
**Age（years）**				0.504	0.293
⩽50	49	31	18		
＞50	166	114	52		
**Tumor site**				1.341	0.158
colon	142	92	50		
Rectum	73	53	20		
**Tumor size**				0.2	0.382
＜5cm	112	74	38		
⩾5cm	103	71	32		
**Differentiation**				1.81	0.404
Well	13	10	3		
Moderate	150	97	53		
Poor	52	38	14		
**T stage**				0.179	0.914
T1-2	18	12	6		
T3-4	197	133	64		
**Lymph node status**				43.103	0.000
Positive	103	92	11		
Negative	112	53	59		
**Distant metastasis**				3.996	0.034
Yes	22	19	3		
No	193	126	67		
**Vascular invasion**				215.000	0.000
Positive	58	45	13		
Negative	157	100	57		
**Clinical stage**				40.405	0.000
Ⅰ + Ⅱ stage	108	51	57		
Ⅲ + Ⅳ stage	107	94	13		

Supplementary Table S2. Spearman correlation analysis between the expression levels of CSRP2 and Clinic pathologic Features

Variables	CSRP2 expression	
	Spearman correlation	P value
Gender	0.007	0.913
Age	-0.048	0.48
Tumor size	-0.03	0.657
Differentiation	-0.039	0.571
Lymph node status	-0.427	0
Distant metastasis	-0.136	0.046
Clinical stage	-0.392	0

Supplementary Table S3. The Relationship between CSRP2 expression and clinic pathological features

Characteristics	CSRP2 expression				
	n=215	Low	High	x2 value	**P** value
**Tumor size（Ⅰ+Ⅱ stage）**				4.558	0.026
＜5cm	54	25	29		
⩾5cm	54	36	18		
**Tumor size（Ⅲ+Ⅳ stage）**				2.682	0.081
＜5cm	58	49	9		
⩾5cm	49	35	14		
